# Dotinurad restores exacerbated kidney dysfunction in hyperuricemic patients with chronic kidney disease

**DOI:** 10.1186/s12882-024-03535-9

**Published:** 2024-03-15

**Authors:** Hoichi Amano, Seiji Kobayashi, Hiroyuki Terawaki

**Affiliations:** 1 Department of Nephrology, Teikyo University Chiba Medical Center, Chiba, Japan; 2 Clinical Laboratory Department, St. Luke’s International University, Tokyo, Japan

**Keywords:** Chronic kidney disease, Kidney dysfunction, Uric acid, Hyperuricemia, Dotinurad

## Abstract

**Background:**

In this study, we aimed to clarify the beneficial effects of urate-lowering treatment with the novel agent dotinurad on renal function in patients with chronic kidney disease (CKD) and hyperuricemia (HUA).

**Methods:**

Thirty-five patients with CKD (mean age 65.4 ± 14.8 years, 23 men) diagnosed with HUA were recruited. Changes in eGFR before and after dotinurad administration were assessed. Patients first underwent a 3-month observation period and then 3 months treatment with dotinurad.

**Results:**

During the observation period, mean eGFR (mL/min/1.73 m^2^) declined significantly. The baseline eGFR was 31.8 ± 16.4 and the serum urate level (sUA, mg/dL) was 8.1 ± 1.7. During the treatment period, eGFR recovered to 36.5 ± 17.5 and sUA decreased to 6.7 ± 1.0. The increase in eGFR after dotinurad administration was correlated with a decrease in sUA (*R* = 0.375, *p* = 0.0263).

**Conclusion:**

Dotinurad administration to patients with CKD and HUA appears to be beneficial in restoring kidney function. Dotinurad may represent a potential medication for the prevention of kidney function decline caused by HUA.

## Introduction

Hyperuricemia (HUA) is a noncommunicative disease whose prevalence has rapidly increased since World War II [1]. HUA causes gout, including gouty arthritis, and also increases the risk of kidney dysfunction or chronic kidney disease (CKD) [2–4]. For instance, we previously demonstrated through a cohort study that HUA increases the risk of CKD occurrence 3.99-fold among the general population [4]. Therefore, HUA is considered to be a cause of CKD.

On the other hand, the necessity of urate-lowering therapy for CKD patients with HUA is uncertain. In other words, whether HUA is an aggravating factor for CKD or not is unclear.

In this study, we aimed to investigate whether urate-lowering therapy would improve the course of kidney function in patients with CKD and HUA using dotinurad, a novel selective urate reabsorption inhibitor (SURI) that selectively inhibits the urate transporter 1 (URAT1 / SLC22A12), as an antihyperuricemic agent.

## Methods

### Study design, participants, and intervention (Fig. 1)

**Fig. 1:**
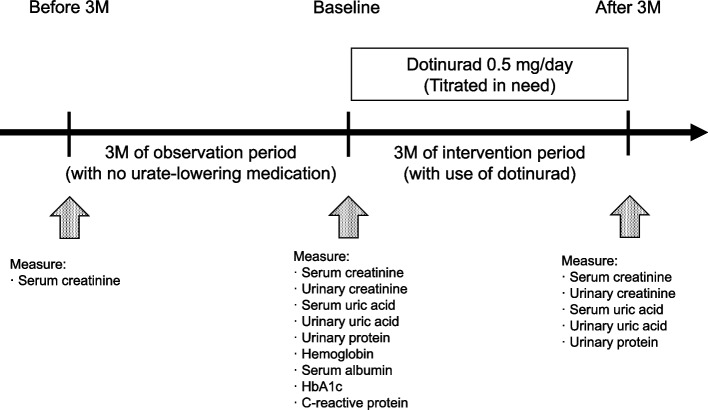
Schematic presentation of present study

The study participants were outpatients who visited the Division of Nephrology at Teikyo University Chiba Medical Center Hospital between April 2021 and November 2021 and met the criteria for CKD [5] and HUA [serum uric acid (sUA) level > 7 mg/dL, symptomatic gout, or both] [6]. Patients with a history of systemic diseases such as malignancy and collagen diseases were excluded.

The patients first underwent a 3-month observation period and then 3 months of dotinurad treatment. To be specific, after a 3-month observation period before intervention, 0.5 mg/day of dotinurad was administered once a day. In most cases, the dose of dotinurad was increased to 1.0 mg/day to achieve sUA levels < 6.0 mg/dL [6]. Other medications – including irbesartan (1 case) and losartan (2 cases) that have a urate-lowering effect and hydrochlorothiazide (1 case) that have a urate-elevating effect—were not changed during the study period.

### Data collection

Demographic, clinical, and laboratory data were reviewed. Levels of sUA, serum creatinine (sCr), hemoglobin, serum albumin, serum cholinesterase, urinary protein, urinary creatinine, and urinary uric acid were measured at our hospital using standardized reagents and methods. The degree of proteinuria was quantified using the spot urine protein-creatinine ratio (UPCR, g/gCr). The fractional excretion of uric acid (FEUA) was also calculated before dotinurad administration and 3 months after.

Kidney function was expressed as the estimated glomerular filtration rate (eGFR), which was determined using the following formula proposed by the Japanese Society of Nephrology [7]:

$${\text{eGFR}}\left({\text{mL}}/{\text{min}}/1.73\mathrm{ m}2\right)=194\times {{\text{sCr}}}^{-1.094}\times {{\text{Age}}}^{-0.287}\times 0.739 \left(\mathrm{if\, female}\right).$$

### Statistical analysis

Continuous data are expressed as means ± standard deviation or medians with 25th and 75th percentiles, and categorical data are expressed as percentages. Changes in various parameters before and after dotinurad administration were determined using paired Student’s t-test.

eGFR-pre represents the difference in eGFR at the start of treatment (baseline) and before 3 months of treatment, and ΔeGFR-post represents the difference in eGFR after 3 months of treatment and at baseline. Changes in eGFR before and after dotinurad administration (difference between eGFR-pre and ΔeGFR-post) were also determined using paired Student’s t-test. Stratified analyses were performed on the basis of gender and age.

Differences with a *p*-value < 0.05 were considered statistically significant. All statistical analyses were performed using EZR Version 1.33 (Saitama Medical Center, Jichi Medical University, Saitama, Japan), a graphical user interface for R (R Foundation for Statistical Computing, Vienna, Austria). More precisely, it is a modified version of the R commander, which is designed to add statistical functions that are frequently used in biostatistics [8].

## Results

Baseline characteristics and laboratory data of the participants are presented in Table 1. At starting of treatment, mean age was 65.4 ± 14.8 years and 71.4% of the patients were male. Mean eGFR was 31.7 ± 16.4 and mean sUA was 8.1 ± 1.7. Of the 35 participants, 17 patients classified as CKD stage G3, 13 as CKD stage G4, and 5 as CKD stage G5. None of the participants complained of adverse effects of dotinurad during the study period.

**Table 1:** Patients' profile at starting of treatment

Characteristics	
Age, years	65.4 ± 14.8
Male, n (%)	25 (71.4)
Body mass index, kg/m2	23.4 ± 5.2
Systolic blood pressure, mmHg	133 ± 18
Diastolic blood pressure, mmHg	77 ± 15
Primary CKD, n	
Chrinic glomerulonephritis	17
Diabetic kidney disease	8
Nephrosclerosis	5
Gout kidney	5
Laboratory data	
Hemoglobin, g/dL	12.8 ± 2.4
Serum albumin, g/dL	4.0 ± 0.5
HbA1c, % (NGSP)	6.0 ± 0.8
Serum creatinine, mg/dL	1.9 ± 0.87
eGFR-cre, mL/min/1.73 m^2^	31.8 ± 16.4
30 or higher (%)	17 (48.6%)
< 30 (%)	18 (51.4%)
Serum uric acid, mg/dL	8.1 ± 1.7
C-reactive protein, mg/dL	0.50 ± 0.53
Urinary protein, g/gCr	1.1 ± 1.2
Fractional excretion of uric acid, %	7.56 ± 4.68

Changes in sUA and various parameters other than eGFR before and after dotinurad use (baseline and after 3 Mo) are shown in Table 2. After 3 months of dotinurad administration, the mean sUA decreased significantly from 8.1 ± 1.7 to 6.7 ± 1.0. Regarding FEUA, no significant difference was observed between baseline and after 3 Mo, suggesting that the decrease in sUA via inhibition of URAT1 by dotinurad probably had already reached equilibrium.

**Table 2:** Changes in various parameters before and after using dotinurad

	Baseline		After 3 M		*p*-value
	Mean	SD	Mean	SD	
Serum creatinine, mg/dL	1.90	0.87	1.73	0.82	< 0.001
Serum uric acid, mg/dL	8.1	1.7	6.7	0.99	< 0.001
Urinary protein, g/gCr	1.1	1.2	1.2	1.2	0.2
Fractional excretion of uric acid, %	7.56	4.68	7.35	3.56	0.8

**Fig. 2:**
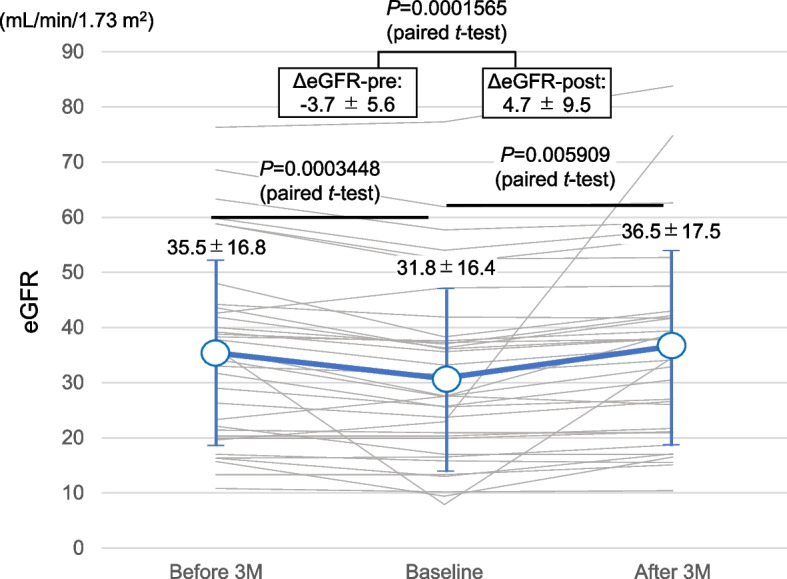
Changes in eGFR during the observation and intervention periods. During 3 months of observation, the mean eGFR decreased significantly. During 3 months of intervention or after the administration of urate-lowering treatment using dotinurad, the mean eGFR increased significantly

Figure 2 presents a comparison of eGFR values before 3 months, at baseline, and after 3 months. During 3 months of observation, mean eGFR declined significantly from 35.5 ± 16.8 to 31.8 ± 16.4, suggesting that kidney dysfunction was progressive in these patients. Additionally, during 3 months of intervention, mean eGFR increased from 31.8 ± 16.4 to 36.5 ± 17.5, suggesting that kidney function was recovered by urate-lowering treatment using dotinurad, and accordingly, mean ΔeGFR-pre showed a negative value (-3.7 ± 5.6), whereas mean ΔeGFR-post showed a positive value (4.7 ± 9.5), and the difference between them was statistically significant. Such beneficial effects of dotinurad administration on kidney function were observed regardless of gender, age, eGFR at starting of treatment, or primary CKD diagnosis (Table 3).

**Table 3:** Changes in eGFR before and after using dotinurad: stratified analysis regarding gender, age, CKD stage and primary disease

	n	ΔeGFR-pre		ΔeGFR-post		*p*-value
		Mean	SD	Mean	SD	
All	35	-3.7	5.6	4.7	9.5	< 0.001
Gender						
Male	25	-4.6	6	3.2	5.3	0.001
Female	10	-1.5	3.7	8.6	15.6	0.061
Age						
65 or higher	23	-4	3.1	4.6	10.8	< 0.001
< 65	12	-3.3	8.3	4.9	7.3	0.075
CKD stage at starting of treatment (mL/min/1.73 m^2^)						
G3	17	-3.9	3.6	2.6	2.1	< 0.001
G4	13	-2.0	3.8	6.2	14.1	0.043
G5	5	-7.7	11.8	8.0	10.8	0.195
Primary disease						
Gout kidney	5	-4.9	1.6	3.2	1.5	0.001
Diabetic kidney disease	5	-3.3	2.5	0.8	1.9	0.047
Nephrosclerosis	8	-2.1	2.6	2.8	1.9	0.016
Chronic glomerulonephritis	17	-4.3	7.6	7.2	13.3	0.010

**Fig. 3:**
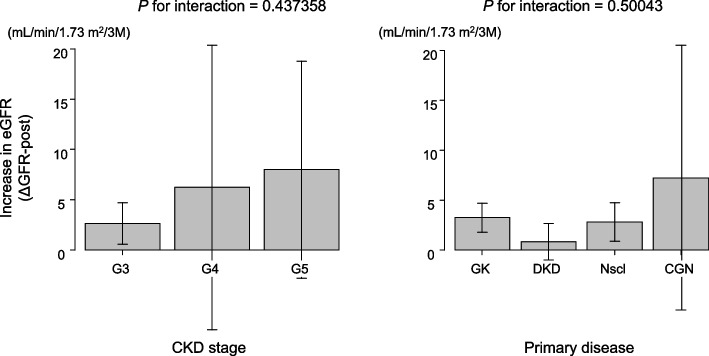
Increase in eGFR after dotinurad administration (ΔeGFR-post) stratified by CKD stage and primary disease. The difference in CKD stage or primary disease did not affect the nephroprotective effect of dotinurad. GK, gout kidney; DKD, diabetic kidney disease; Nscl, nephrosclerosis; CGN, chronic glomerulonephritis

To evaluate whether the difference in kidney function or primary disease may influence the nephroprotective effect of dotinurad, we performed a multiway analysis of variance, in which ΔeGFR-post was adopted as the objective variable and CKD stage or primary disease as explanatory variable. The result is shown in Fig. 3. Neither CKD stage nor primary disease influenced the degree of improvement in kidney function after the administration of dotinurad.

**Fig. 4:**
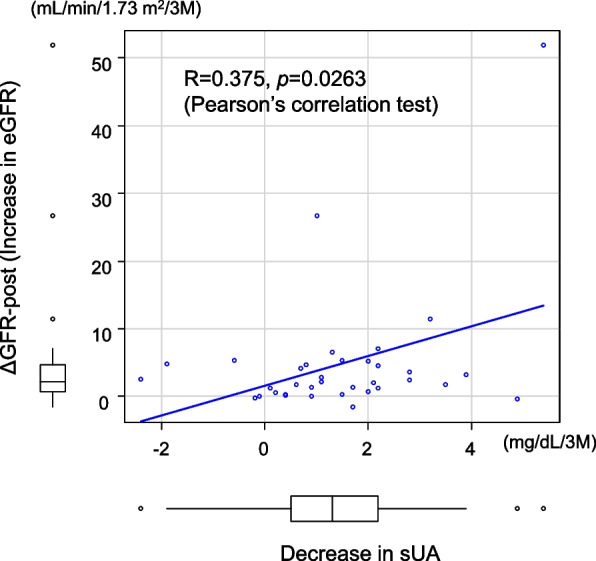
Significant relationship between decreased sUA and ΔeGFR-post (increase in eGFR) after dotinurad administration

Figure 4 shows the relationship between the decrease in sUA and GFR-post (increase in eGFR) during 3 months of intervention. A significant positive correlation was observed between them, suggesting that the improvement in kidney function after dotinurad usage was caused not by a profound pleiotropic effect of this drug but by the decrease in the reabsorption of urate per se.

## Discussion

In the present single-center study of CKD patients with HUA, changes in kidney function before and after administration of dotinurad were investigated. Kidney function declined during the 3 months of observation and then increased after dotinurad administration. This result suggested two possible explanations.

1. HUA could contribute to the exacerbation of kidney dysfunction in patients with CKD, at least in certain settings.
2. Dotinurad restored kidney dysfunction caused by HUA.

The number of patients with CKD has been increasing worldwide, and the disease is estimated to affect 200 million individuals worldwide [9]. Furthermore, the number of patients with CKD is expected to increase. CKD creates a considerable burden and is recognized as an important problem for both individuals and society. CKD is a risk factor for not only end-stage kidney disease (ESKD) but also cardiovascular disease, which is the main cause of death worldwide [10, 11]. In addition, worldwide medical expenses associated with hemodialysis for ESKD are estimated to increase to US$1,000 billion within the next 10 years [12]. Therefore, establishing an effective strategy for CKD remission is an important public and national health issue.

Accumulating evidence has shown that HUA is associated with CKD progression [2–4]; however, most trials to clarify whether urate-lowering treatment can attenuate the decline in renal function in patients with CKD have failed to achieve these goals [13–15]. Although such studies adopted xanthine oxidoreductase inhibitor as a urate-lowering drug, adaptation of SURI as a urate-lowering drug might demonstrate reno-protective effects on progressive kidney damage related to HUA. Histologic findings of renal injury directly related to HUA include deposition of monosodium urate monohydrate in the renal interstitium [16–18], and SURI might prevent the passive inflow of urate from the internal lumen of proximal collecting tubule to the renal interstitum via URAT1 and the tubular cell matrix. This possibility is supported by the clear relationship between decreased sUA and increased eGFR observed in the present study (Fig. 4).

Of course, it is important to review diet and lifestyle in hyperuricemia, as in diabetes, which is a lifestyle-related disease such as hyperuricemia. On the other hand, as in diabetes, the nephroprotective effect of dietary and lifestyle changes in hyperuricemia is unclear. Our results suggest that SURI, which acts directly on the renal proximal tubules of the kidney, has a distinct nephroprotective effect, as do SGLT2 inhibitors, which also act directly on the renal proximal tubules.

The result of present study, however, should be interpreted with caution, for patients with CKD with apparent HUA – like present study population—may not teem in the real world, and the beneficial effect of dotinurad is supposed to be limited to patients with apparent HUA. This study had certain limitations. First, it is unclear whether the findings could be generalized to other ethnic or age groups because the subjects analyzed were all outpatients of one hospital, and the possibility of sampling bias cannot be denied. Second, the number of participants was small, preventing us from performing a stratified analysis. Third, the present study was a single-arm observational study, and we only compared post-treatment and pre-treatment phases and did not compare changes in kidney function between patients who did and did not use dotinurad. Fourth, pathological findings from renal biopsies were not examined in this study. Fifth, the reduction of deposed monosodium urate monohydrate in the renal interstitium through appropriate diagnostic images such as dual energy CT was not demonstrated in the present study. To overcome these limitations, prospective randomized studies with larger numbers of patients are required.

In conclusion, in the present study, the use of dotinurad in CKD patients with HUA appeared to be beneficial for preserving kidney function, and these results further indicate that this novel SURI might be a potential key medication for preventing kidney function decline in CKD patients with HUA.

## Data Availability

The data presented in this study are available on request from the corresponding author.

## Abbreviations

CKD: Chronic kidney disease
HUA: Hyperuricemia
eGFR: Estimated glomerular filtration rate
SURI: Selective urate reabsorption inhibitor
URAT: Urate transporter
SLC: Solute carrier
sUA: Serum uric acid
sCr: Serum creatinine
UPCR: Urine protein-creatinine ratio
ESKD: End-stage kidney disease
